# miR-195-5p Suppresses the Proliferation, Migration, and Invasion of Oral Squamous Cell Carcinoma by Targeting TRIM14

**DOI:** 10.1155/2017/7378148

**Published:** 2017-10-24

**Authors:** Tong Wang, Yipeng Ren, Ruixun Liu, Juntao Ma, Yueyi Shi, Lei Zhang, Rongfa Bu

**Affiliations:** ^1^Medical College, Nankai University, Tianjin 300070, China; ^2^Department of Oral and Maxillofacial Surgery, Chinese PLA General Hospital, Beijing 100853, China; ^3^Department of Oral and Maxillofacial Surgery, Tianjin Stomatology Hospital, Tianjin 300041, China

## Abstract

MicroRNAs (miRNAs) play an essential role in tumor biological processes through interacting with specific gene targets. The involvement of miR-195-5p in cell proliferation, invasion, and migration has been demonstrated in several cancer cell lines, while its function in oral squamous cell carcinoma (OSCC) remains unclear. Here we find that miR-195-5p expression is lower in OSCC than in nontumor tissues, while its overexpression in cell lines can lead to the promotion of apoptosis and the reduction of cell growth, migration, and invasion. Moreover, we identify the tripartite motif-containing protein (TRIM14) as a target of miR-195-5p. Therefore, we reason that the tumor suppressor role of miR-195-5p in OSCC is dependent on the interaction with TRIM14.

## 1. Introduction

Oral squamous cell carcinoma (OSCC) is a common head and neck cancer with poor prognosis due to recurrence [1]. Despite the progress of treatments, which include surgery, radiotherapy, and chemotherapy, in the control of oral cancer primary tumor, the 5-year survival rate of OSCC patients was still less than 50% in the last 30 years because of high recurrence rate and mortality [2, 3]. Thus, in order to promote an effective treatment of OSCC, an understanding of the molecular mechanism of OSCC proliferation, invasion, and metastasis, all of which underlie recurrence, is needed.

MicroRNAs (miRNAs) are a class of small, noncoding RNA molecules, approximately 22 nucleotides in length. They regulate gene expression at the posttranscriptional or translational level by binding to complimentary sequences in the 3′-untranslated regions (UTRs) of target mRNAs and affect the biological processes of tumors [4–8]. Multiple miRNAs, such as miR-145, miR-338, and miR-433, have been shown to participate in OSCC physiological and pathological processes, including cell proliferation, apoptosis, migration, metabolism, and differentiation [9–11]. Several reports have observed the reduction of miR-195-5p expression in tumors [12–14]. Indeed, Jia et al. suggested that the overexpression of miR-195-5p could have application in the inhibition of the development and progression of tongue squamous cell carcinoma [15]. However, the mechanism and target gene of miR-195-5p in OSCC remain unknown.

Over 80 protein members of the tripartite motif-containing (TRIM) protein family have been found in the human genome so far, and most of them play significant biological roles comprising innate immunity, transcription, and apoptosis [16–19]. TRIM14 is a recently identified gene located on chromosome 9q22. It is characterized by the existence of a B-box, a coiled coil, and a PRYSPRY domain at the C-terminus, while lacking N-terminal RING domain often found in TRIM proteins. Early reports have shown TRIM14 to be involved in many biological processes by way of NF-*κ*B signaling [20–22].

In our study, we profile the expression patterns of miR-195-5p in OSCC tissues and cell lines. Our findings suggest that miR-195-5p works as a tumor suppressor by downregulating the expression of TRIM14 in OSCC.

## 2. Materials and Methods

### 2.1. Tissue Samples

All OSCC tissues and matched adjacent nontumor samples were obtained from 40 patients who have undergone surgery at Chinese PLA General Hospital (Beijing, China). None of the patients received radiotherapy, chemotherapy, or other special treatment. The tissue samples were frozen in liquid nitrogen for further extraction of RNAs. This study was approved by the Ethics Committee of Chinese PLA General Hospital. Informed consent and approval were obtained from all patients. Clinicopathological characteristics of these patients were summarized in Table 1.

### 2.2. Cell Culture and Transfection

The human OSCC cell lines Tca83 and Cal27 were supplied by the Central Laboratory, Peking University School of Stomatology. The cells were maintained in DMEM supplemented with 10% fetal calf serum (Gibco, Los Angeles, CA, USA) and cultured in a humidified chamber at 37°C and 5% CO_2_. Tca83 or Cal27 cells were transfected with miR-195-5p mimic, miR-195-5p inhibitor, or miR-195-5p scramble (which served as the negative control) using Lipofectamine 2000 (Invitrogen, Carlsbad, CA, USA) according to the manufacturer's instruction.

### 2.3. Extraction of RNA and Quantitative Real-Time PCR

TRIzol reagent was used for the extraction of total RNA from the cell lines and tissues. Complementary DNA was synthesized using the PrimeScript™ RT reagent kit (TaKaRa, Dalian, China). TRIM14 mRNA and miR-195-5p were detected by qPCR using the SYBR Green Kit (TaKaRa, Dalian, China). The control genes U6 snRNA and *β*-actin were used for normalization. The primers were designed and synthetized by GenePharma. The expression data was analyzed using the 2^−ΔΔCt^ method and all experiments were carried in triplicate.

### 2.4. Cell Proliferation (MTT Assay)

Cells after transfection were cultured in 96-well plates at 5000 cells/well for 5 days and then added with 15 *μ*l of MTT (5 mg/ml) to each well. The cells were inoculated for 4 h and the reaction was terminated with 150 *μ*l DMSO. Absorbance was measured at 490 nm using a microplate reader. Experiments were performed in triplicate.

### 2.5. Cell Cycle and Apoptosis Assay

For cell cycle analysis, cells after transfection were harvested, washed, and then fixed with 70% ethanol for 24 hours at −20°C. Afterwards, the cells were stained with propidium iodide (50 *μ*g/ml) containing RNase (0.1 *μ*g/ml). Samples were then incubated for 30 min at room temperature and were detected on a FACSCanto II instrument and analyzed by FlowJo. For apoptosis analysis, cells after transfection were harvested, washed with PBS, and similarly analyzed with Annexin V-FITC and propidium iodide (BD Pharmingen, San Diego, CA, USA).

### 2.6. Transwell Assay

To examine the migration and invasion of cells after transfection, Transwell chambers were coated without or with Matrigel, respectively, on the upper chamber. The bottom chambers were filled with 10% FBS in DMEM. After 24 hours, the cells from the upper chamber were collected with a cotton swab and stained with 0.5% crystal violet. Cells from five random fields were counted using a light microscope.

### 2.7. Luciferase Assay

The 3′-UTR of TRIM14 reporter was created containing one putative miR-195-5p targeting site and one randomly scrambled sequence inserted into the pGL3 vector (Promega, Madison, WI, USA) predicted not to be a target of miR-195-5p. Tca83 and Cal27 cells were cotransfected with the wild-type or mutant plasmid and miR-195-5p or negative control. Following 48 h cell growth, luciferase activity was determined with the Dual-Luciferase Reporter System (Promega, Madison, WI, USA).

### 2.8. Western Blot Analysis

Cells after transfection were harvested and extracted with RIPA. Proteins were resolved by SDS-page on a 10% Bis-Tris gel and then electrotransferred onto PVDF at 60 V for 1.5 h. The membranes were blocked in 5% milk and then probed with primary antibodies against TRIM14 and *β*-actin (Santa Cruz Biotechnology, Dallas, TX, USA.) at 4°C overnight. Then the membranes were incubated with secondary HRP-linked antibodies for 2 h at room temperature and detected by electrochemiluminescence kit. Bands intensity was quantified by ImageJ software.

### 2.9. Statistical Analysis

All numerical data were expressed as mean ± SD from triplicate experiments. Comparisons between groups were performed by Student's two-tailed* t*-test using SPSS 21.0 software. The correlation between miR-195-5p expression and clinicopathological characteristics of OSCC patients was analyzed with *χ*^2^   test or Fisher's exact test. The correlation between miR-195-5p and TRIM14 expression was examined by Spearman's correlation. *P* values less than 0.05 were considered statistically significant.

## 3. Results

### 3.1. miR-195-5p and TRIM14 Are Inversely Expressed in OSCC Cell Lines and Tissues

**Figure 1:**
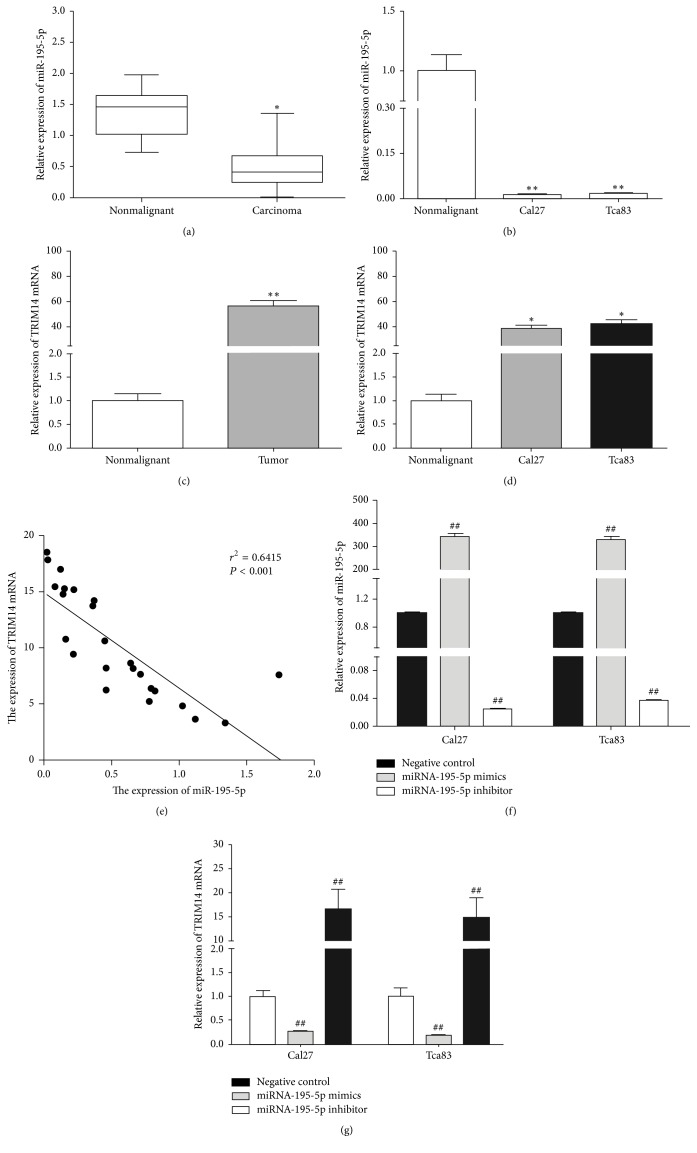
*miR-195-5p and TRIM14 expression in OSCC tissues and cells. *Transcript levels were quantified by qPCR. qRT-PCRs for miR-195-5p have been performed with specific primers for mature miRNA in OSCC tissues (a) and cell lines (b). TRIM14 mRNA expression in OSCC tissues (c) and cell lines (d) was quantified by qRT-PCRs. ^*∗*^*P* < 0.05 and ^*∗∗*^*P* < 0.01 versus nonmalignant group. Correlation of miR-195-5p and TRIM14 expression in OSCC tissues was analyzed by Spearman's correlation analysis (e). The expressions of miR-195-5p (f) and TRIM14 (g) in Tca83 and Cal27 cell lines were quantified by qRT-PCRs after transfection with miR-195-5p mimics and inhibitors, respectively. ^##^*P* < 0.01 versus negative control.

By real-time PCR, the expression of miR-195-5p in OSCC tissues was compared to the corresponding adjacent nontumor samples. The results showed that the expression of miR-195-5p was significantly lower in OSCC tissues (Figure 1(a)). According to the median value, we defined 21 patients with low miR-195-5p expression and 19 patients with high expression of miR-195-5p. As shown in Table 1, we found that the low level of miR-195-5p expression was associated with pathological differentiation grade (*P* = 0.034) and STNMP stage (*P* = 0.024). In addition, downregulation of miR-195-5p was also observed in two OSCC cell lines, Tca83 and Cal27 cells (Figure 1(b)). Concomitantly, we found that the expression levels of TRIM14 mRNA were notably higher in both OSCC tissues and cell lines (Figures 1(c) and 1(d)). As the statistical analysis showed, we found that there existed a reverse correlation between miR-195-5p and TRIM14 expression in OSCC tissues (Figure 1(e)). In order to test whether miR-195-5p had an effect on TRIM14 expression, Tca83 and Cal27 cells were transfected with either miR-195-5p mimic, miR-195-5p inhibitor, or miR-195-5p scramble, which served as a negative control. As shown in Figure 1(f), the transfection of miR-195-5p mimic produced over 300-fold expression levels of miR-195-5p over the negative control. Similarly, appreciable reduction of miR- 195-5p levels was demonstrated with the transfection of the miR-195-5p inhibitor compared to the negative control (Figure 1(f)). When the TRIM14 levels were examined, the upregulation of miR-195-5p using the transfected mimic significantly reduced the expression levels of TRIM14 levels for both cell lines. Conversely, the reduction of miR-195-5p resulted in the increase of TRIM14 levels (Figure 1(g)).

### 3.2. miR-195-5p Suppresses OSCC Cell Proliferation, Invasion, and Migration in Cell Cultures

**Figure 2:**
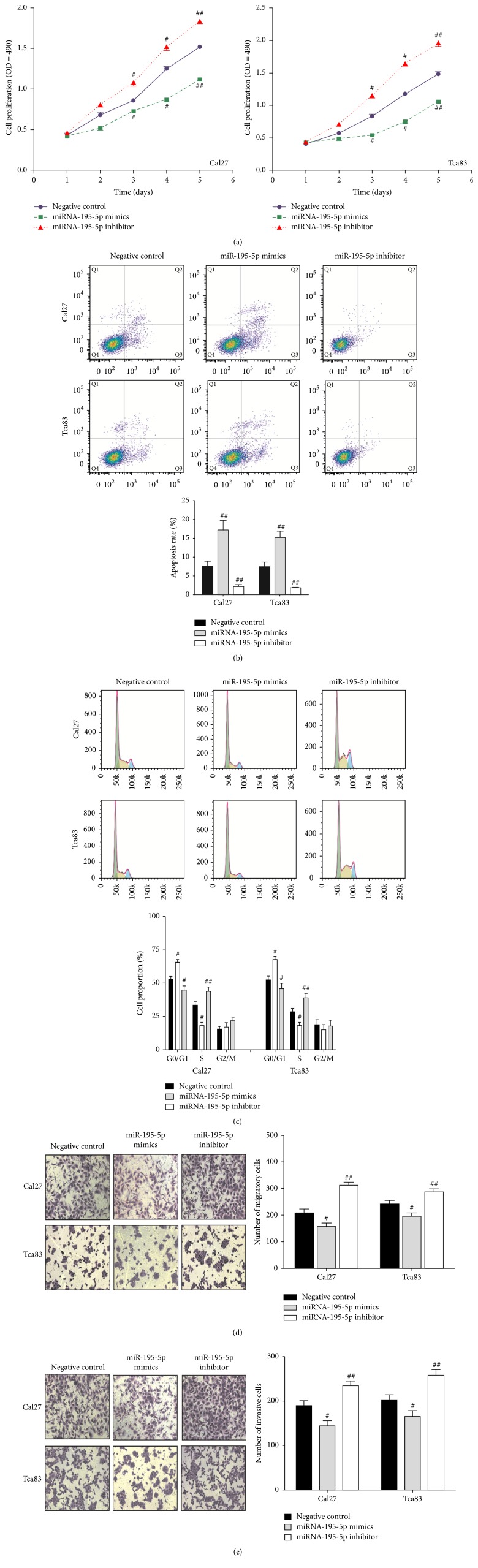
*Overexpression of miR-195-5p inhibited cell proliferation, migration, and invasion of OSCC. *To analyze the cell growth activity, we performed MTT assay at 48 h after transfection with miR-195-5p mimics or inhibitor (a). miR-195-5p contributed to the apoptosis of Tca83 and Cal27 cell lines (b). The percentages of cells in three cell phases of Tca83 and Cal27 cell lines revealed that miR-195-5p may increase the percentage of cells in G0/G1 phase (c). Transwell migration (d) and invasion assay (e) on Tca83 and Cal27 cells infected with miR-195-5p mimics and inhibitor was performed. ^#^*P* < 0.05 and ^##^*P* < 0.01 versus negative control.

In order to shed light on the function of miR-195-5p in OSCC, we first investigated the effect of miR-195-5p on the proliferation of OSCC cells. Cal27 and Tca83 cells were transfected with miR-195-5p mimic, miR-195-5p inhibitor, or miRNA scramble and assessed for cell proliferation by MTT. In both Cal27 and Tca83 cells, the upregulation of miR-195-5p resulted in decreased cell proliferation in comparison to the negative control, while the downregulation of miR-195-5p promoted cell proliferation (Figures 2(a) and 2(b)).

Next, the impact of miR-195-5p on apoptosis and cell cycle was examined. As demonstrated by flow cytometry, the upregulation of miR-195-5p led to increased levels of apoptosis in comparison to the negative control for both Cal27 and Tca83 cells, while the downregulation of miR-195-5p resulted in decreased levels of apoptosis (Figure 2(d)). Specifically, there was an increase in cell populations in the G0/G1 phase and a decrease in cell populations in the S phase observed for both cell lines when miR-195-5p was overexpressed (Figure 2(c)). By contrast, downregulation of miR-195-5p resulted in the reverse trend (Figure 2(c)).

Finally, the effect of miR-195-5p on cell migration and invasion was investigated by the Transwell migration assay. Both Cal27 and Tca83 cells showed decreased migratory and invasive properties when miR-195-5p was upregulated compared to the negative control (Figures 2(d) and 2(e)). By contrast, significant migration and invasion were observed when miR-195-5p was downregulated (Figures 2(d) and 2(e)). Taken together, the results support miR-195-5p as a suppressor of proliferation, migration, and invasion in OSCC.

### 3.3. miR-195-5p Directly Downregulates the Expression of TRIM14 in Cell Cultures

**Figure 3:**
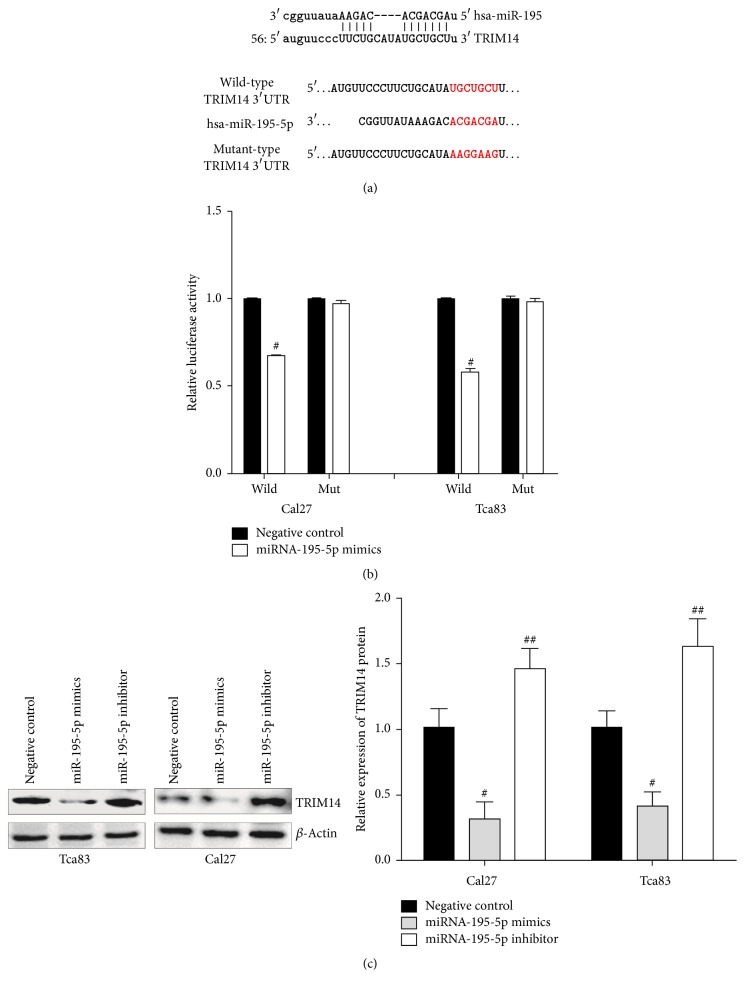
*TRIM14 is a direct target of miR-195-5p.* Sequence alignment of miR-195-5p with the wild-type 3′-UTR of TRIM14 (a). Luciferase assay in Tca83 and Cal27 cells after cotransfection with wt/mut 3′-UTR with miR-195-5p mimics or NC as indicated. 48 hours after transfection, the luciferase activity was detected by using Dual-Luciferase Reporter Assay System according to the protocol (b). The Western blot assay of TRIM14 in Tca83 and Cal27 cells after transfection with miR-195-5p mimics, inhibitor, or NC, respectively (c). ^#^*P* < 0.05 and ^##^*P* < 0.01 versus negative control.

Search for miRNA target by TargetScan (Ref. Agarwal 2015; eLife, 4:e05005) and miRanda (Bino et al.; PLoS Biol., 2004) predicted the 3′UTR of TRIM14 as a potential target of miR-195-5p (Figure 3(a)). In order to test whether the 3′UTR of TRIM14 was indeed a target of miR-195-5p, a reporter construct containing the 3′UTR sequence of TRIM14 was constructed and evaluated by a luciferase assay (Figure 3(a)). As is shown in Figure 3(b) for both Cal27 and Tca83 cells, the upregulation of miR-195-5p resulted in ~50% decrease in luciferase activity compared to the negative control, while the mutant 3′UTR of TRIM14, predicted not to be a target of miR-195-5p, showed no response. In accordance with the luciferase assay, TRIM14 protein levels were reduced >50% upon upregulation of miR-195-5p (Figure 3(b)). By comparison, the suppression of miR-195-5p resulted in ~50% increase in TRIM14 protein levels (Figure 3(b)). Taken together, these experimental results are consistent with TRIM14 as a target of miR-195-5p.

### 3.4. Restoration of TRIM14 Abolishes the Effects of miR-195-5p Induction in OSCC

**Figure 4:**
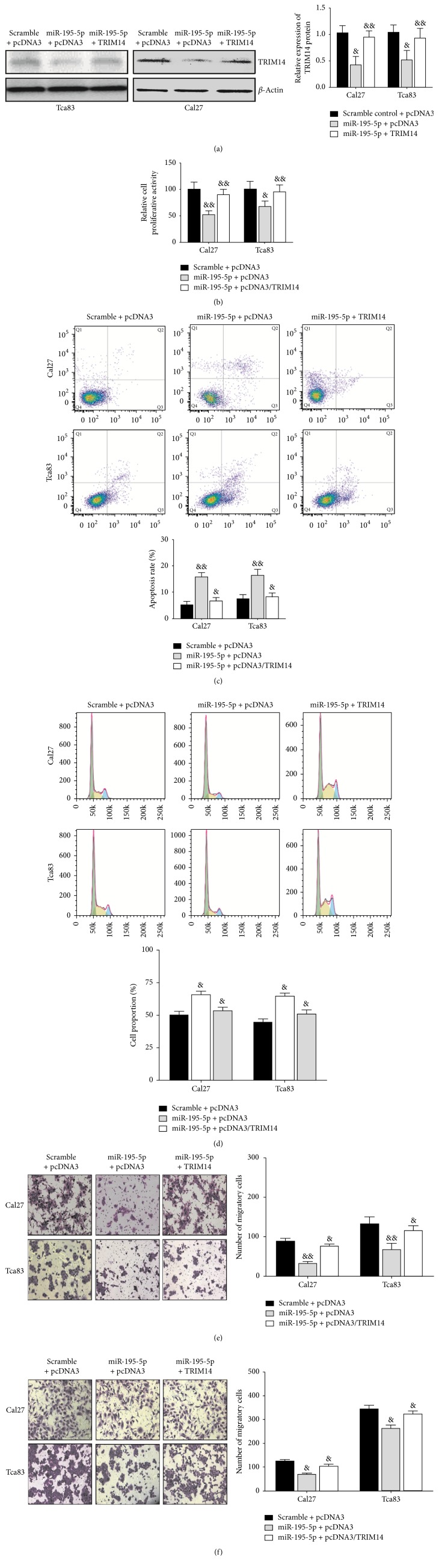
*Ectopic expression of TRIM14 reversed the effects of miR-195-5p on OSCC cells. *Tca83 and Cal27 cells were transfected with miR-195-5p along with TRIM14 plasmid lacking the 3′UTR or NC, and Western blot assay was conducted 48 hours after transfection (a). MTT assay (b), apoptosis assay (c), cell cycle assay (d), invasion assay (e), and migration assay (f) of miR-195-5p-expressing cells transfected with pcDNA3 or TRIM14. ^&^*P* < 0.05 and ^&&^*P* < 0.01 versus scramble + pcDNA3 group.

In order to provide further evidence of the link between miR-195-5p and TRIM14, Tca83 and Cal27 cells were cotransfected with a TRIM14 construct lacking the 3′UTR and miR-195-5p, therefore replenishing the miR-195-5p-depleted endogenous TRIM14 reservoir with exogenous TRIM14 (Figure 4(a)). Consistent with the observed downregulation of miR-195-5p (Figures 2(a) and 2(b)), both cell proliferation (Figure 4(b)) and apoptosis (Figure 4(c)) were now decreased. Also, there was a decline in cell populations in the G0/G1 phase (Figure 4(d)). Finally, the overexpression of TRIM14 recovered the invasive and migration properties of Tca83 and Cal27 cells (Figures 4(e) and 4(f)), which are consistent with a decrease in miR-195-5p levels (Figures 2(d) and 2(e)). Taken together, the compensatory effect of an exogenous TRIM14 in restoring phenotypes invoked by miR-195-5p upregulation strongly supports TRIM14 as the target for miR-195-5p.

## 4. Discussion

Supporting lines of evidence have demonstrated the importance of miRNAs toward biological characteristics of various cancers, such as cell proliferation, apoptosis, invasion, and migration [23–28]. As such, miRNAs have gained interest as therapeutic targets. The involvement of miRNAs in the initiation and progression of oral cancer has been reported in recent years [29–31]. Recent findings revealed the involvement of several miRNAs (miR-21, miR-221, miR-455, etc.) with respect to the tumorigenesis and progression of oral cancer [32–37].

miR-195-5p belongs to the miR-15 family, which is associated with various cancers, including thyroid cancer, hepatocellular carcinoma, glioblastoma, and breast cancer [38]. In these findings, miR-195-5p displayed low expressions in tumors and acts as a key negative regulator. Given this, we speculated that miR-195-5p might also serve a tumor suppressor role in OSCC. In our study, miR-195-5p was downregulated in oral cancer cell lines and tissue samples. By upregulating the expression of miR-195-5p, we were able to show the ability of miR-195-5p to suppress cell proliferation, which was further corroborated by the observation of G0/G1 cell arrest. Additionally, cell migration and invasion were suppressed, while apoptosis was promoted.

Since miRNAs can act as either oncogenes or tumor suppressors through interaction with specific targets [39, 40], we identified TRIM14 as a potential target of miR-195-5p. Su et al. found that TRIM14 was upregulated in tongue squamous cell carcinoma (TSCC), and its overexpression contributed to the progression of TSCC by activating the NF-*κ*B signaling pathway [41]. In this study, miR-195-5p is shown to affect the expression of TRIM14 at both the transcript and protein levels. Moreover, the ability of exogenous TRIM14 to restore miR-195-5p-depleted TRIM14 reservoir, all together, provides compelling evidence that TRIM14 is a bona fide target of miR-195-5p. Therefore, miR-195-5p likely suppresses proliferation, migration, and invasion in OSCC through interaction with TRIM14. As such, the targeting of the miR-195-5p/TRIM14 interaction could serve as a new therapeutic design strategy for the treatment of OSCC.

## Figures and Tables

**Table 1:** Association between miR-195 expression and clinicopathological characteristics of 40 oral squamous cell carcinoma patients.

Clinical characteristics	Total	miR-195 expression		*P* value
		Low	High	
All	40	21	19	
Gender				0.370^#^
Male	26	15	11	
Female	14	6	8	
Age				0.218^#^
<60	17	7	10	
≥60	23	14	9	
Lesion site				0.477^*∗*^
Buccal mucosa	6	3	3	
Tongue	13	9	4	
Mouth floor	6	2	4	
Gingiva	15	7	8	
Pathological differentiation grade				0.034^*∗*^
Well	11	2	9	
Moderate	22	14	8	
Poor	7	5	2	
STNMP stage				0.024^*∗*^
I	11	2	9	
II	22	13	9	
III	6	5	1	
IV	1	1	0	

^#^ *χ* ^2^ test; ^*∗*^Fisher's exact test.
