# Expression of Concern on “miR-195-5p Suppresses the Proliferation, Migration, and Invasion of Oral Squamous Cell Carcinoma by Targeting TRIM14”

**DOI:** 10.1155/2019/8739375

**Published:** 2019-07-18

**Authors:** BioMed Research International

BioMed Research International would like to express concern with the article titled “miR-195-5p Suppresses the Proliferation, Migration, and Invasion of Oral Squamous Cell Carcinoma by Targeting TRIM14” published in October 2017 [1] due to figure duplication, as raised on PubPeer. In the first panel of Figure 4(f), part of Cal27 (Scramble + pcDNA3) appears to be the same as part of Cal27 (miR-195-5p + TRIM14). This duplication was included after the first revision, when the authors added data for the Cal27 cell line.

We asked the authors to explain this and provide the underlying images and raw data for all the figures. They said that they introduced this error when they used PowerPoint to put one image of the Cal27 (Scramble + pc DNA3) group into the Cal27 (miR-195-5p + TRIM14) group by mistake, though they could not provide the correct images as their laptop was damaged in 2017 and they lost the data and images for this experiment. A member of the editorial board recommended that the authors repeat the experiment to correct the panel for Figure 4(f). BioMed Research International will reach a final decision regarding the article once the authors have had an opportunity to replicate these results.
