# Corrigendum to “miR-195-5p Suppresses the Proliferation, Migration, and Invasion of Oral Squamous Cell Carcinoma by Targeting TRIM14”

**DOI:** 10.1155/2022/9865894

**Published:** 2022-05-12

**Authors:** Tong Wang, Yipeng Ren, Ruixun Liu, Juntao Ma, Yueyi Shi, Lei Zhang, Rongfa Bu

**Affiliations:** ^1^Medical College, Nankai University, Tianjin 300070, China; ^2^Department of Oral and Maxillofacial Surgery, Chinese PLA General Hospital, Beijing 100853, China; ^3^Department of Oral and Maxillofacial Surgery, Tianjin Stomatology Hospital, Tianjin 300041, China

In the article titled “miR-195-5p Suppresses the Proliferation, Migration, and Invasion of Oral Squamous Cell Carcinoma by Targeting TRIM14” [1], a figure duplication was identified in Figure 4(f) as noted in an earlier Expression of Concern [2]. With the agreement of the editorial board, the authors have repeated the migration and invasion experiments and provided a revised Figure 4 as below:

## Figures and Tables

**Figure 1:**
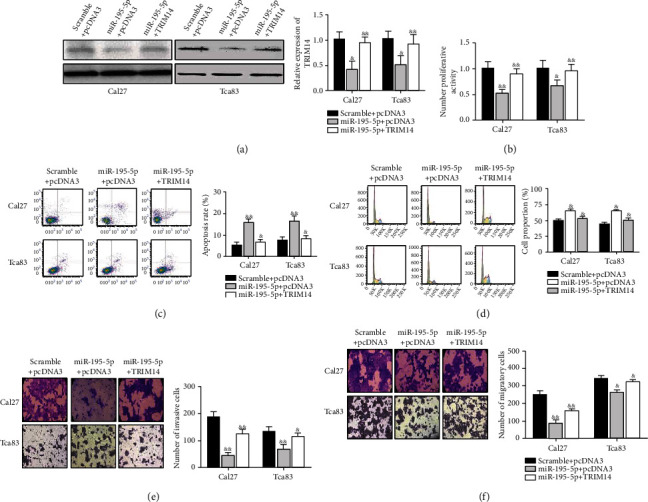
Ectopic expression of TRIM14 reversed the effects of miR-195-5p on OSCC cells. Tca83 and Cal27 cells were transfected with miR-195-5p along with TRIM14 plasmid lacking the 3′UTR or NC, and Western blot assay was conducted 48 hours after transfection (a). MTT assay (b), apoptosis assay (c), cell cycle assay (d), invasion assay (e), and migration assay (f) of miR-195-5p-expressing cells transfected with pcDNA3 or TRIM14. ^&^*P* < 0.05 and ^&&^*P* < 0.01 versus scramble+pcDNA3 group.
