# Supplementary material for: Corrigendum to “miR-195-5p Suppresses the Proliferation, Migration, and Invasion of Oral Squamous Cell Carcinoma by Targeting TRIM14”
Source: Biomed Res Int. 2022 May 12;2022:9865894. doi: 10.1155/2022/9865894 (PMC9119784; doi:10.1155/2022/9865894)
Supplement: Supplementary Materials — The raw data for Figure 4 can be found in the Supplementary files. [file 9865894.f1.zip › 9865894.f1/Figure and data.pptx]

## Slide 1
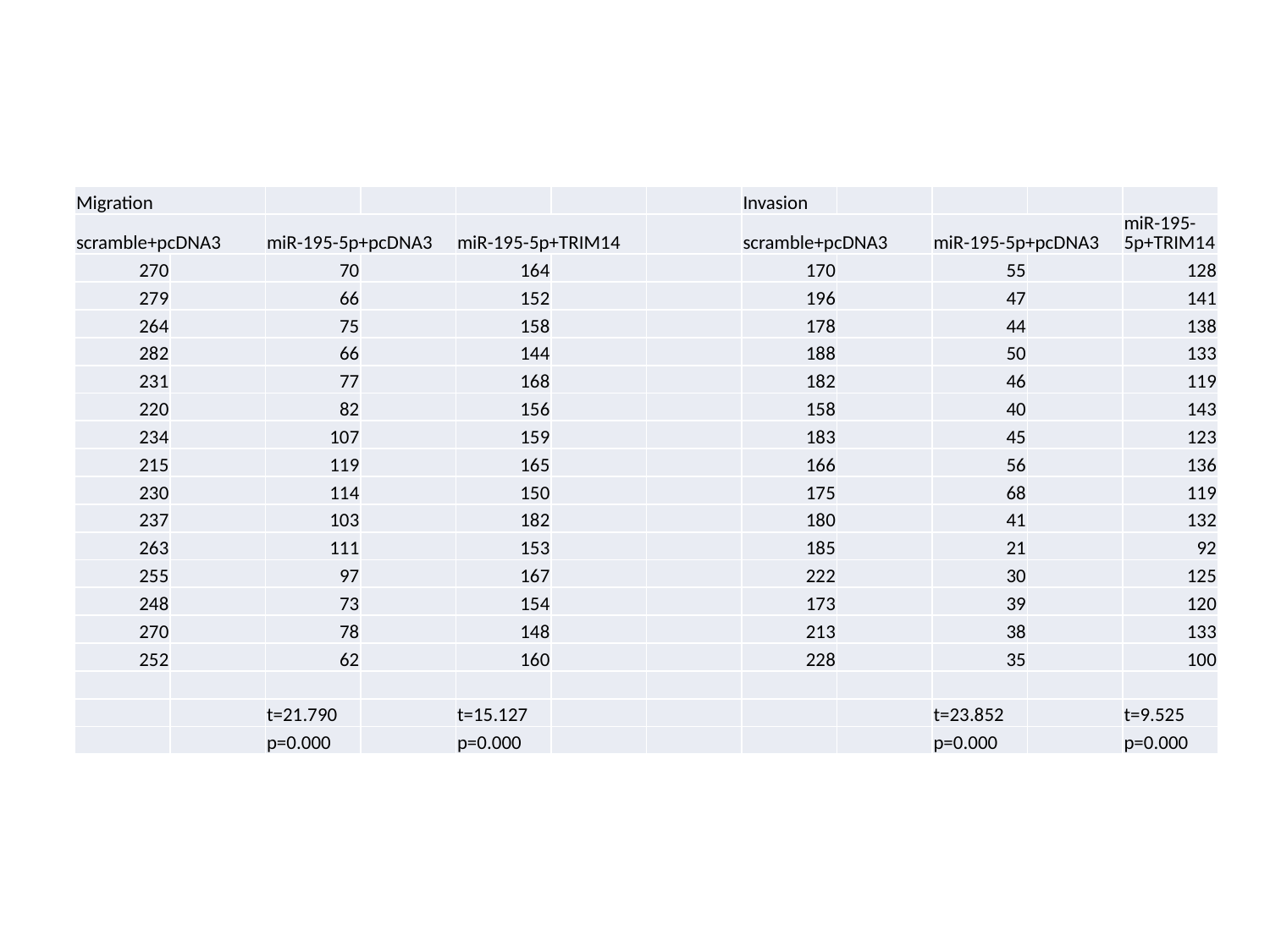

| Migration | | | | | | | Invasion | | | | |
| --- | --- | --- | --- | --- | --- | --- | --- | --- | --- | --- | --- |
| scramble+pcDNA3 | | miR-195-5p+pcDNA3 | | miR-195-5p+TRIM14 | | | scramble+pcDNA3 | | miR-195-5p+pcDNA3 | | miR-195-5p+TRIM14 |
| 270 | | 70 | | 164 | | | 170 | | 55 | | 128 |
| 279 | | 66 | | 152 | | | 196 | | 47 | | 141 |
| 264 | | 75 | | 158 | | | 178 | | 44 | | 138 |
| 282 | | 66 | | 144 | | | 188 | | 50 | | 133 |
| 231 | | 77 | | 168 | | | 182 | | 46 | | 119 |
| 220 | | 82 | | 156 | | | 158 | | 40 | | 143 |
| 234 | | 107 | | 159 | | | 183 | | 45 | | 123 |
| 215 | | 119 | | 165 | | | 166 | | 56 | | 136 |
| 230 | | 114 | | 150 | | | 175 | | 68 | | 119 |
| 237 | | 103 | | 182 | | | 180 | | 41 | | 132 |
| 263 | | 111 | | 153 | | | 185 | | 21 | | 92 |
| 255 | | 97 | | 167 | | | 222 | | 30 | | 125 |
| 248 | | 73 | | 154 | | | 173 | | 39 | | 120 |
| 270 | | 78 | | 148 | | | 213 | | 38 | | 133 |
| 252 | | 62 | | 160 | | | 228 | | 35 | | 100 |
| | | | | | | | | | | | |
| | | t=21.790 | | t=15.127 | | | | | t=23.852 | | t=9.525 |
| | | p=0.000 | | p=0.000 | | | | | p=0.000 | | p=0.000 |

## Slide 2
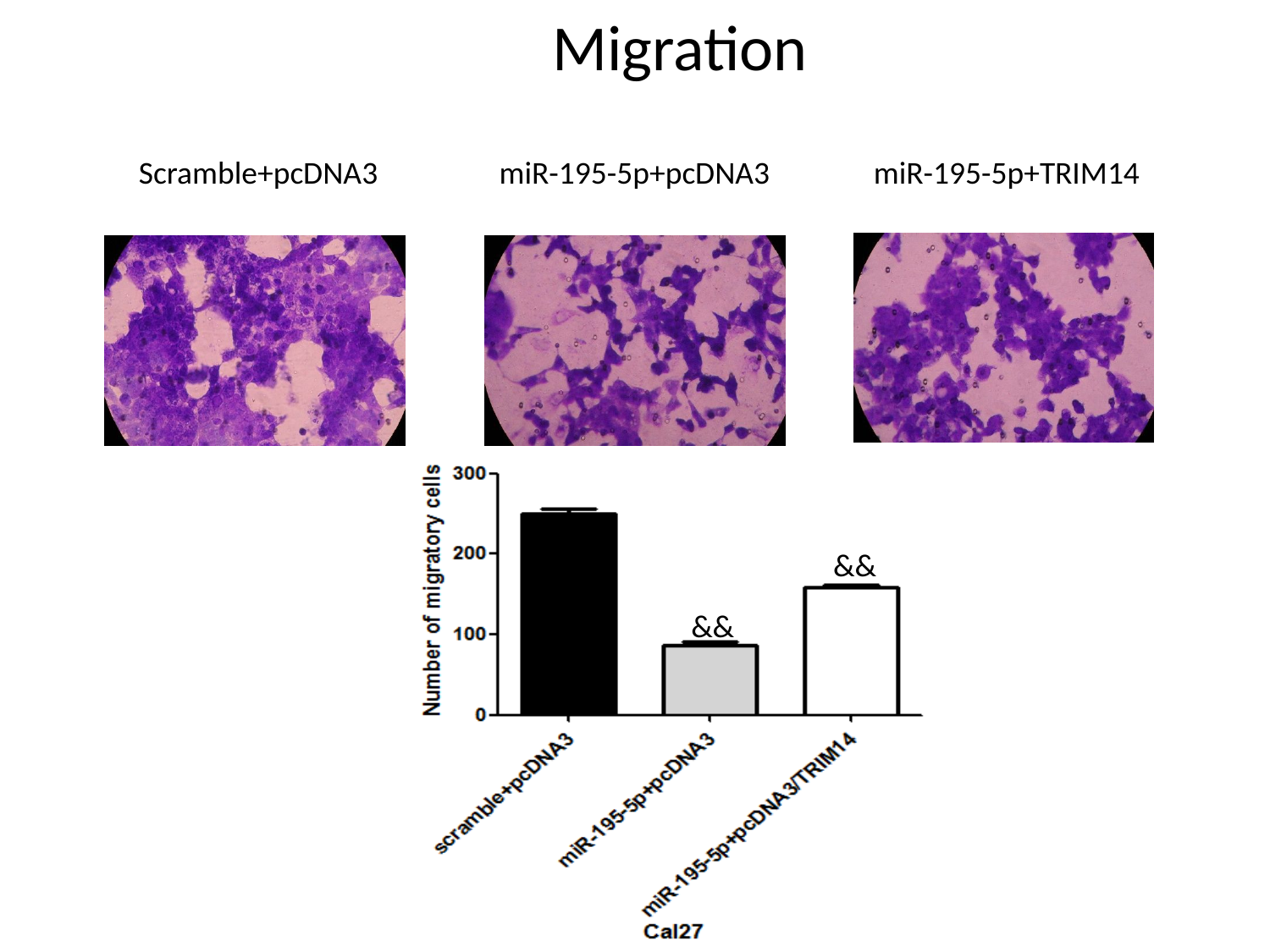

# Migration
Scramble+pcDNA3
miR-195-5p+pcDNA3
miR-195-5p+TRIM14
&&
&&

## Slide 3
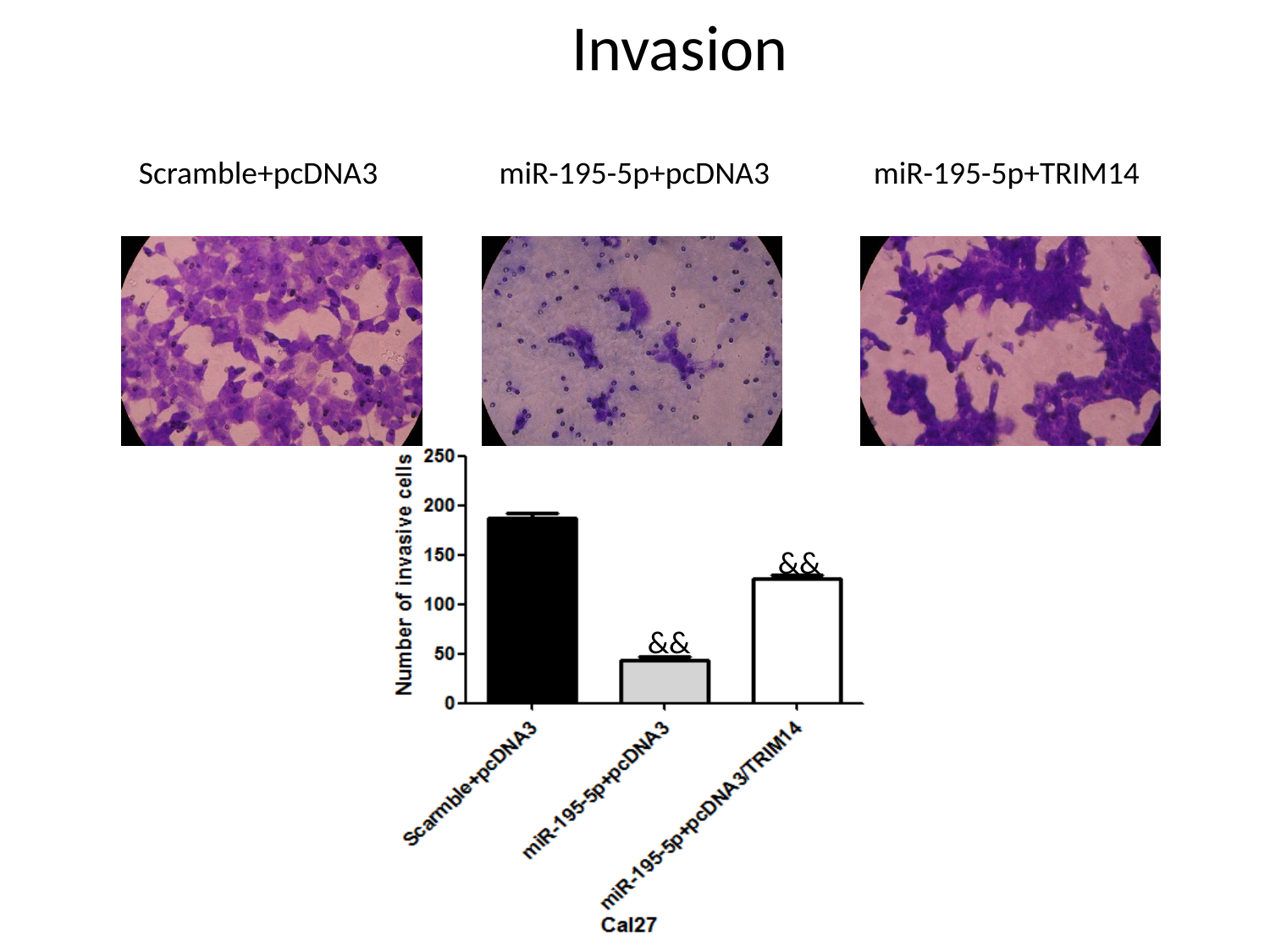

# Invasion
Scramble+pcDNA3
miR-195-5p+pcDNA3
miR-195-5p+TRIM14
&&
&&
